# Supplementary material for: Microwave Near-Field Dynamical Tomography of Thorax at Pulmonary and Cardiovascular Activity
Source: Diagnostics (Basel). 2023 Mar 9;13(6):1051. doi: 10.3390/diagnostics13061051 (PMC10047846; doi:10.3390/diagnostics13061051)
Supplement: Supplementary file 1 [file diagnostics-13-01051-s001.zip › animation.pptx]

## Slide 1
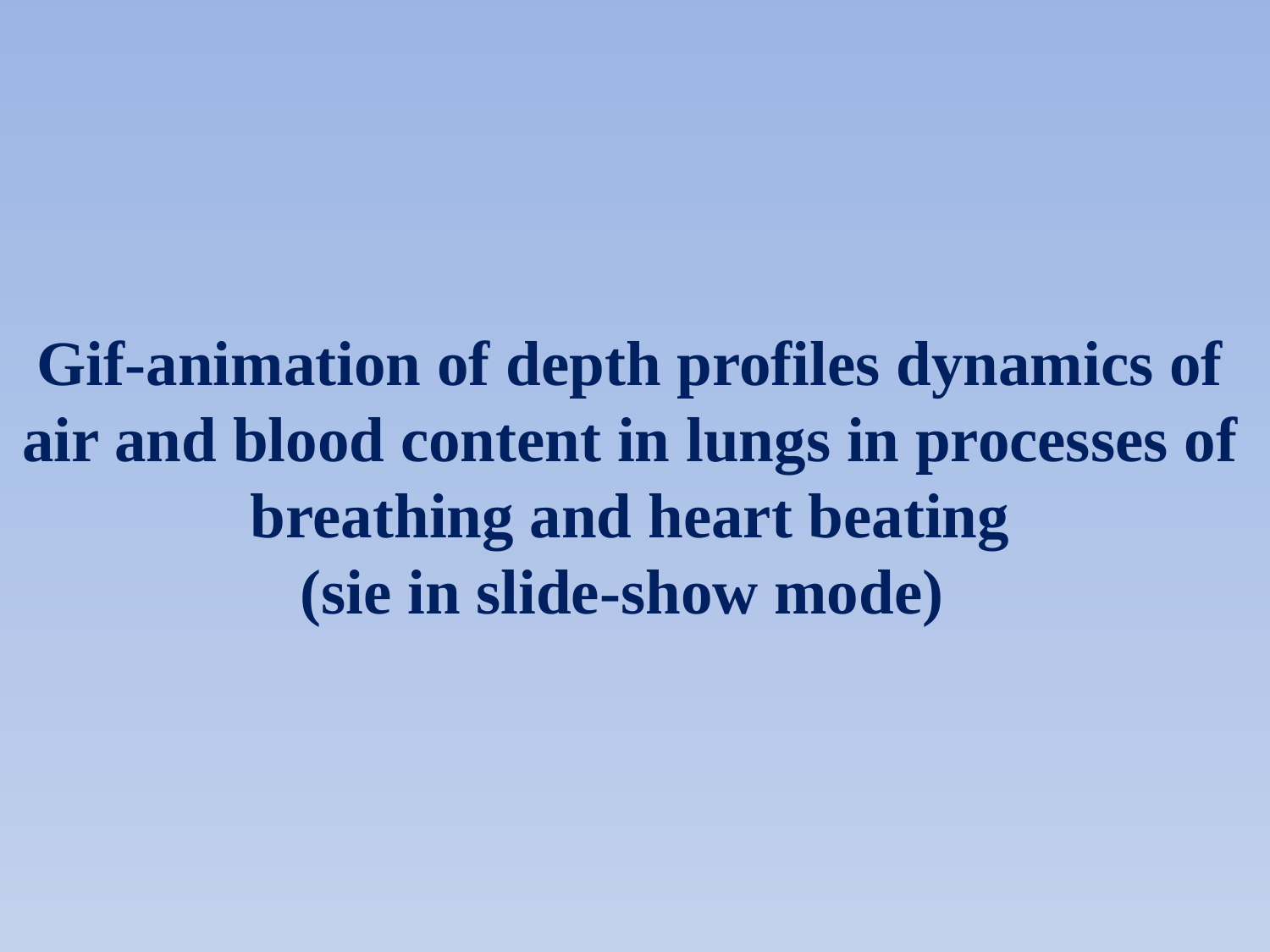

Gif-animation of depth profiles dynamics of air and blood content in lungs in processes of breathing and heart beating
(sie in slide-show mode)

## Slide 2
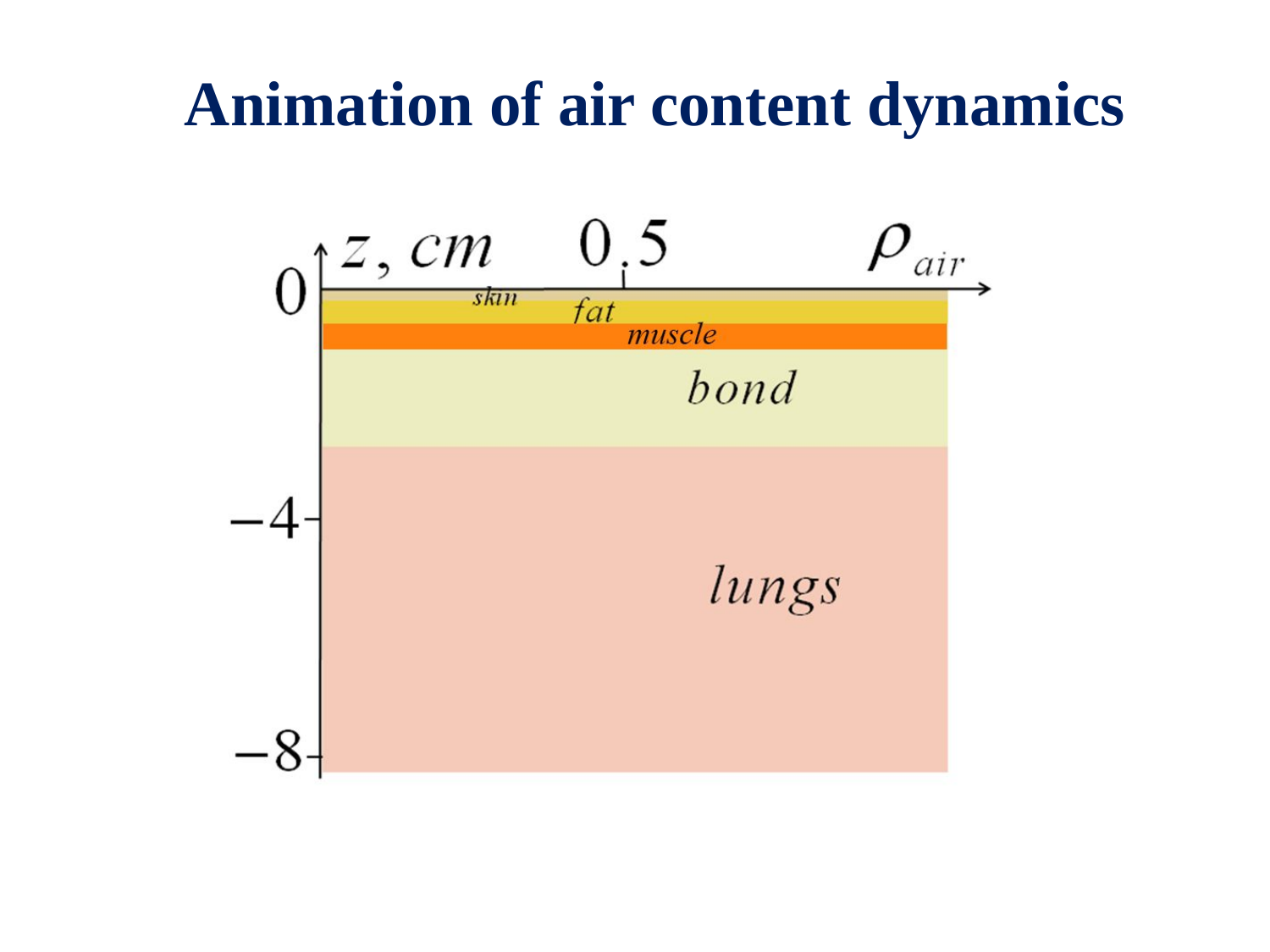

Animation of air content dynamics

## Slide 3
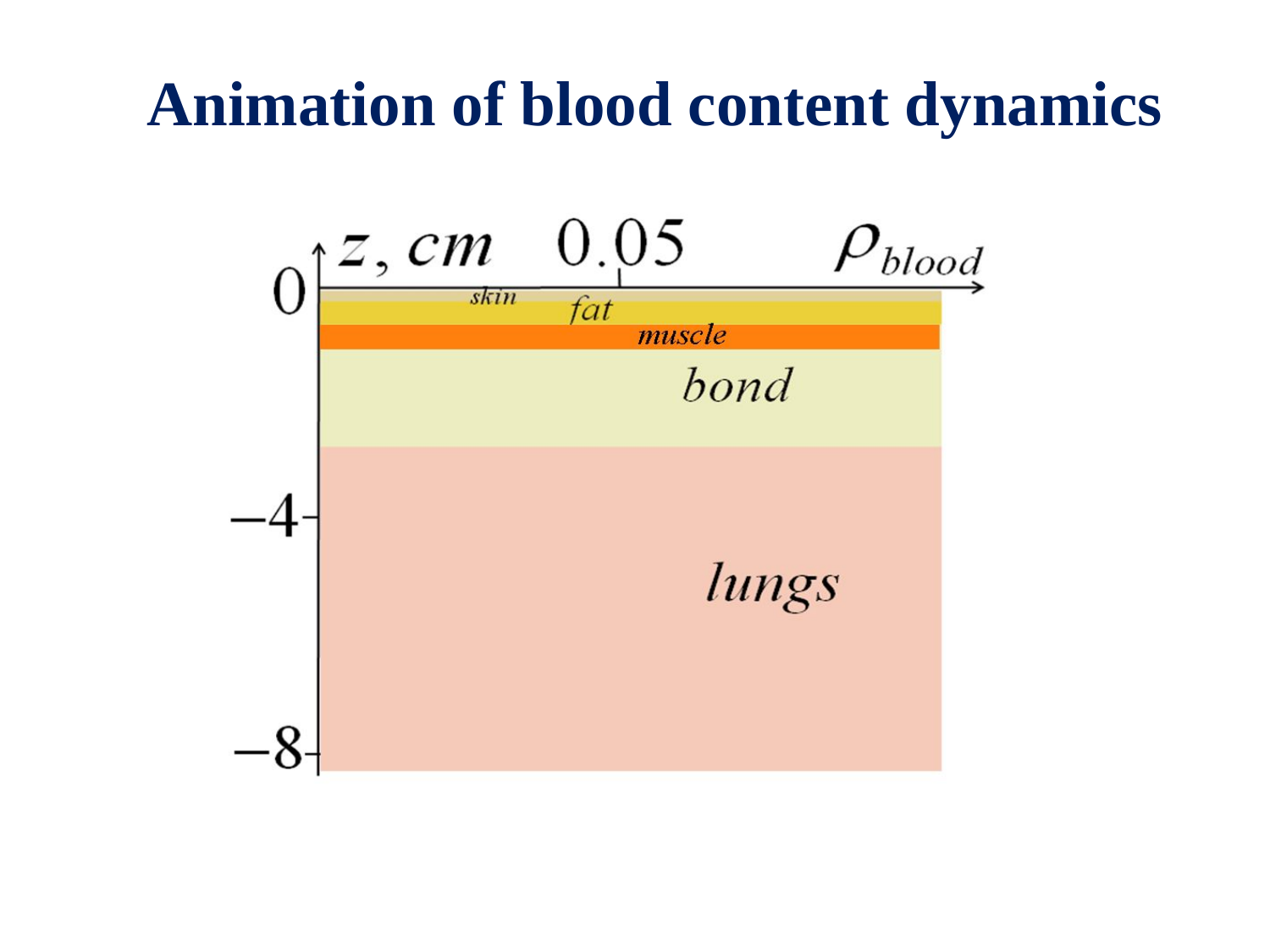

Animation of blood content dynamics
