# Supplementary material for: Microwave Near-Field Dynamical Tomography of Thorax at Pulmonary and Cardiovascular Activity
Source: Diagnostics (Basel). 2023 Mar 9;13(6):1051. doi: 10.3390/diagnostics13061051 (PMC10047846; doi:10.3390/diagnostics13061051)
Supplement: Supplementary file 1 [file diagnostics-13-01051-s001.zip › Ethics Committee.pdf]

федеральное государственное бюджетное учреждение
«Национальный медицинский исследовательский центр
сердечно-сосудистой хирургии имени А.Н. Бакулева»
Министерства здравоохранения Российской Федерации
121552, г. Москва, [illegible]

Ethics Committee report

№ 4 meeting of the ethical commission of the FSBI "National Medical Research Center for Cardiovascular Surgery named after A.N. Bakulev "of the Ministry of Health of Russia dated December 2, 2020 No.
Agenda:
Consideration of the issue of approving the continuation of research in the experiment and on volunteers according to the approved research methodology for "Monitoring of changes in the parameters of biological tissues of the respiratory and cardiovascular systems by the novel technology of electromagnetic tomography."

Submitted documents:
1) Research protocol;
2) Patient questionnaire for registration of electromagnetic fields of the body;
3) Informed consent to participate in the study.

The meeting took place in the premises of the FSBI "National Medical Research Center for Cardiovascular Surgery named after A.N. Bakulev "of the Ministry of Health of Russia at the address: Moscow, Rublevskoe shosse, 135.
Resolved:
The Ethics Committee approves the continuation of research in the experiment and on volunteers on the approved research method of electromagnetic tomography "Monitoring of changes in the parameters of biological tissues of the respiratory and cardiovascular systems by the novel technology of electromagnetic tomography."

Chairman of the Ethics Committee
Federal State Budgetary Institution
"A. N. Bakulev National Medical Research Center for
Cardiovascular Surgery" Ministry of Health of Russia,

Ph.D. Filatov A.G.

Secretary of the Ethics Commission, Ph.D. Tarashvili E.G.

Для
